# Metagenome-assembled bacterial genomes from benthic microbial mats in ice-covered Lake Vanda, Antarctica

**DOI:** 10.1128/mra.01250-23

**Published:** 2024-04-08

**Authors:** Tyler Powell, Dawn Y. Sumner, Anne D. Jungblut, Ian Hawes, Tyler Mackey, Christen Grettenberger

**Affiliations:** 1 Department of Earth and Planetary Sciences, University of California, Davis, USA; 2 Microbiology Graduate Group, University of California, Davis, USA; 3 Department of Sciences, The Natural History Museum, London, United Kingdom; 4 Coastal Marine Field Station, University of Waikato, Tauranga, New Zealand; 5 Department of Earth and Planetary Sciences, University of New Mexico, Albuquerque, New Mexico, USA; 6 Department of Environmental Toxicology, University of California, Davis, USA; California State University San Marcos, San Marcos, California, USA

**Keywords:** Antarctica, microbial mat, metagenome-assembled genomes

## Abstract

We recovered 57 bacterial metagenome-assembled genomes (MAGs) from benthic microbial mat pinnacles from Lake Vanda, Antarctica. These MAGs provide access to genomes from polar environments and can assist in culturing and utilizing these Antarctic bacteria.

## ANNOUNCEMENT

Most microorganisms, especially extremophilic organisms cannot be cultured easily under *in vitro* laboratory conditions (1, 2). Molecular methodologies like metagenomic shotgun sequencing now provide a culture-independent method of examining extremophilic taxa and the adaptations that they have for life in these extreme environments. They provide a helpful alternative approach to answer questions about the organisms and their community when culturing attempts are unsuccessful. Here, we retrieved 57 metagenome-assembled genomes (MAGs) from microbial pinnacles that were subsampled from benthic mats that grew on the floor of the perennially ice-covered Lake Vanda, Antarctica.

Samples were collected as described previously (3). Briefly, scientific divers sampled microbial mat pinnacles in Lake Vanda in the McMurdo Dry Valleys, Antarctica (−77.529, 161.578) between 8th and 18th December 2013. Microbial mats were subsampled based on color into photosynthetically active green (G) or purple (P) areas and aphotic beige (IB) areas within pinnacles (4). DNA was extracted in the field from multiple green, purple, and inner beige samples and one un-subsampled “bulk mat” sample. DNA was extracted using the Zymo Soil/Fecal miniprep kit (Zymo Research, USA). Libraries were prepared using the KAPA HyperPrep kit (KAPA Biosystems, USA) and DNA was sequenced using the Illumina HiSeq-2500 1TB platform with 2 × 151 bp paired-end sequencing and quality controlled using the standard Joint Genome Institute protocol as described previously (3). Briefly, reads with four or more “N” bases with average quality less than three or minimum length ≤51  bp or 33% of the full read length and those with 93% identity to masked human, dog, cat, mouse, and common contaminants were removed using BBDuk (version 37.36) within BBTools (5). Metagenomes were assembled using MEGAHIT v1.9.6 with a minimum contig length of 1500 (6). Reads were mapped to the assembly using Bowtie2 v1.2.2 and samtools v1.7 (7, 8). A depth file was generated using the jgi_summarize_bam_contig_depths command in MetaBAT v.2.12.1 (9). Bins were generated using metaBAT using a minimum contig size of 2,500 bp. The completeness and contamination of the bins were calculated using CheckM v1.0.7 (10). Bins > 90% complete with less than 5% contamination were retained. Average nucleotide identity (ANI) between bins was calculated using the anvi-compute-genome-similarity command in Anvi’o v6.2 using fastANI (11, 12). Bins sharing >98% ANI was considered to be the same taxon and the bin with the highest completeness was selected as the representative MAG for that taxon. Representative MAGs were classified using GTDB-tk v1.7.0 (13). Previously published MAGs were removed (3, 14, 15). Genome coverage was calculated using coverM (https://github.com/wwood/CoverM) and genomes were annotated using the NCBI Prokaryotic Genome Annotation Pipeline (16). Default parameters were used unless otherwise noted.

Metagenomes contained 177–229 million reads which assembled into 179,000–223,000 contigs (Table 1). We retrieved 57 MAGs from 20 bacterial phyla. The median contig number for MAGs was 264. The average size and GC content were 4.296 Mb and 52.828%, respectively. Average completeness was 96.41% and contamination was 1.38%. Coverage ranged from 11.1× to 111.3×. There were 2,130–6,778 protein-encoding genes (Table 1).

**TABLE 1:** Summary of metagenome statistics and MAG properties^*a*^

	Metagenome statistics					Genome statistics									
Site	Accession	Raw read pairs (millions)	Quality controlled read pairs (millions)	Contigs	N50	Accession	Genome name	Classification	Total length (Mb)	GC (%)	Contigs	Complete-ness (%)	Contam-ination (%)	Coverage (×)	Protein-encoding genes (total)
Bulk Mat	SRX3539175	208	207	179,859	4156	SAMN38186868	BulkMat.140	Bacteria; Bacteroidota; Bacteroidia; Chitinophagales; Saprospiraceae; JABWAJ01	5.56	49.33	409	97.78	1.01	50.0	4,494
						SAMN38186869	BulkMat.37	Bacteria; Pseudomonadota; Alphaproteobacteria; Caulobacterales; TH1-2; Terricaulis	3.7	62.78	136	98.62	1.54	23.1	3,852
						SAMN38186870	BulkMat.75	Bacteria; Zixibacteria; MSB-5A5; GN15; FEB-12; JACRAV01	2.7	48.5	82	98.84	0	44.0	2,246
						SAMN38186871	BulkMat.82	Bacteria; Chloroflexota; Anaerolineae; Aggregatilineales; A4b; GCA-2794515	3.9	47.52	509	96.18	1.82	15.4	3,595
MP5G1	SRX3539176	95	94	213,763	4255	SAMN38186872	MP5G1.132	Bacteria; Bacteroidota; Bacteroidia; Chitinophagales; Saprospiraceae; JABWAJ01	5.76	49.38	437	97.78	2.27	24.6	4,673
						SAMN38186873	MP5G1.138	Bacteria; Spirochaetota; Leptospirae; Leptospirales; Leptospiraceae; Leptospira_A	4.11	35.7	37	100	0	44.6	3,834
						SAMN38186874	MP5G1.179	Bacteria; Chloroflexota; Chloroflexia; Chloroflexales; Roseiflexaceae	6.13	54.9	757	95.91	1.89	12.1	5,489
						SAMN38186875	MP5G1.50	Bacteria; Bacteroidota; Bacteroidia; AKYH767; B-17BO; UBA2475	3.53	36.14	190	97.44	2.66	28.9	3,021
MP5IB2	SRX3539179	103	102	217,012	4652	SAMN38186876	MP5IB2.11	Bacteria; Bdellovibrionota; Bdellovibrionia; JABDDW01; JABDDW01	3	39.57	70	95.48	2.68	17.6	2,935
						SAMN38186877	MP5IB2.114	Bacteria; Bacteroidota; Bacteroidia; NS11-12g; SHWZ01	3.5	36.82	205	96.43	0.63	20.4	2,970
						SAMN38186878	MP5IB2.151	Bacteria; Planctomycetia; Pirellulales; Lacipirellulaceae; Bythopirellula	4.77	56.21	204	97.63	0	42.4	4,053
						SAMN38186879	MP5IB2.172	Bacteria; Bacteroidota; Bacteroidia; Chitinophagales; Saprospiraceae; JADKGY01	3.92	44.25	298	95.42	0.41	18.5	3,141
						SAMN38186880	MP5IB2.194	Bacteria; Bacteroidota; Rhodothermia; Rhodothermales;	5.14	62.43	304	95.9	2.19	21.4	4,229
						SAMN38186881	MP5IB2.201	Bacteria; Bdellovibrionota; Bdellovibrionia; Bdellovibrionales; SG-bin7	3.26	42.33	111	99	1.79	17.7	3,273
						SAMN38186882	MP5IB2.37	Bacteria; Bacteroidota; Bacteroidia; NS11-12g; UKL13-3; B1	3.59	37.51	149	95.24	1.59	23.9	3,020
						SAMN38186883	MP5IB2.67	Bacteria; Gemmatimonadota; Gemmatimonadota; Gemmatimonadales; GWC2-71-9; SZUA-320	3.29	67.18	419	95.6	2.2	27.9	3,077
						SAMN38186884	MP5IB2.78	Bacteria; Planctomycetota; Planctomycetia; Planctomycetales; Planctomycetaceae; DSVQ01	7.43	63.99	717	95.51	1.23	15.0	6,251
MP6G1	SRX3539172	101	100	223,185	4583	SAMN38186885	MP6G1.103	Bacteria; Chloroflexota; Anaerolineae; Aggregatilineales; A4b; OLB15	5.21	59.04	383	98.18	0.91	25.8	4,619
						SAMN38186886	MP6G1.12	Bacteria; Planctomycetota; Phycisphaerae; Phycisphaerales; SM1A02; JAEUIT01	4.3	64.28	281	95.8	2.84	20.6	3,691
						SAMN38186887	MP6G1.168	Bacteria; Chloroflexota; Anaerolineae; Aggregatilineales; A4b; OLB15	4.44	61.39	335	97.27	0	28.5	4,031
						SAMN38186888	MP6G1.198	Bacteria; Myxococcota; Polyangiia; Palsa-1104_A	5.23	59.6	389	95.81	1.31	19.8	4,590
						SAMN38186889	MP6G1.94	Bacteria; Bacteroidota; Bacteroidia; Flavobacteriales; UA16	3.7	41.41	269	95.61	0	16.1	2,907
MP6IB1	SRX3539177	101	100	182,767	5165	SAMN38186890	MP6IB1.110	Bacteria; Bacteroidota; Ignavibacteria; SJA-28; B-1AR	3.61	37.22	262	95.36	1.09	31.4	3,123
						SAMN38186891	MP6IB1.118	Bacteria; Bacteroidota; Bacteroidia; Chitinophagales; Saprospiraceae; JADJLQ01	4.75	42.75	325	95.17	0.99	23.7	3,981
						SAMN38186892	MP6IB1.130	Bacteria; Pseudomonadales; Alphaproteobacteria; Caulobacterales; Hyphomonadaceae; UBA7672	3.61	64.33	253	97.65	2.03	40.7	3,409
						SAMN38186893	MP6IB1.145	Bacteria; Proteobacteria; Alphaproteobacteria; Dongiales; Rhodospirillaceae	3.76	63.88	143	98.91	0.82	30.5	3,523
						SAMN38186894	MP6IB1.15	Bacteria; Bacteroidota; Bacteroidia; Cytophagales; Cyclobacteriaceae; ELB16-189	3.46	50.16	52	99.45	0.55	18.2	3,064
						SAMN38186895	MP6IB1.21	Bacteria; Pseudomonadota; Gammaproteobacteria; Lysobacterales; SZUA-36; SHZL01	4.04	54.27	175	97.83	2.76	16.6	3,703
						SAMN38186896	MP6IB1.62	Bacteria; Verrucomicrobiota; Verrucomicrobiia	2.76	48.16	132	95.95	1.38	16.0	2,590
						SAMN38186897	MP6IB1.66	Bacteria; Proteobacteria; Alphaproteobacteria; Micropepsales; Micropepsaceae; SZUA-430	3.53	58.5	234	96.35	0	41.9	3,561
						SAMN38186898	MP6IB1.68	Bacteria; Planctomycetota; Planctomycetia; Pirellulales; JAEUIK01	7.29	61.18	534	93.79	4.44	16.2	5,962
						SAMN38186899	MP6IB1.8	Bacteria; Nitrospirota; Nitrospiria; Nitrospirales; Nitrospiraceae; Palsa-1315	2.85	56.68	133	96.76	3.41	111.3	2,896
						SAMN38186900	MP6IB1.81	Bacteria; Bacteroidota; Kapabacteria; Kapabacteriales; Kapabacteriaceae; UBA2333	3.04	50.5	71	95.9	1.09	20.3	2,514
						SAMN38186901	MP6IB1.97	Bacteria; Proteobacteria; Alphaproteobacteria; Caulobacterales; TH1-2; VFBF01	3.33	65.96	240	95.94	1.49	20.3	3,382
MP7G1	SRX3539171	107	106	219,283	4526	SAMN38186902	MP7G1.125	Bacteria; Bdellovibrionota_C; UBA2361; UBA2361	2.96	37.52	483	96.14	3.3	12.4	2,731
						SAMN38186903	MP7G1.130	Bacteria; Planctomycetota; UBA1135; UBA1135; GCA-002686595; JAEUIA01	4.49	64	287	97.85	4.48	26.6	3,736
						SAMN38186904	MP7G1.139	Bacteria; Nitrospirota; Nitrospiria; Nitrospirales; Nitrospiraceae; Palsa-1315	4.32	56.62	282	95.91	3.01	41.7	4,447
						SAMN38186905	MP7G1.168	Bacteria; Verrucomicrobiota; Verrucomicrobiia; Methylacidiphilales; JAAUTS01; JAAUTS01	2.4	47.45	74	97.3	1.69	17.4	2,307
						SAMN38186906	MP7G1.59	Bacteria; Gemmatimonadota; Gemmatimonadetes; Gemmatimonadales; GWC2-71-9	4.17	67.42	403	97.8	4.44	17.8	3,870
MP8IB2	SRX3539178	94	92	205,304	4796	SAMN38186907	MP8IB2.145	Bacteria; Proteobacteria; Alphaproteobacteria; Micropepsales; Micropepsaceae; SZUA-430	5.07	63.43	186	99.78	1.74	30.5	4,930
						SAMN38186908	MP8IB2.79	Bacteria; Candidatus Eisenbacteria; RBG-16-71-46; JABDJR01	3.96	62.82	264	95.6	3.3	39.5	3,429
MP9P1	SRX3539181	116	115	200,348	4604	SAMN38186909	MP9P1.103	Bacteria; Cyanobacteria; Vampirovibrionia; Obscuribacterales; Obscuribacteraceae	8.13	50.27	283	95.73	3.99	24.5	6,547
						SAMN38186910	MP9P1.11	Bacteria; Bacteroidota; Bacteroidia; Chitinophagales; CAIOSU01	4.53	44.3	137	96.55	0.33	40.0	3,885
						SAMN38186911	MP9P1.153	Bacteria; Planctomycetota; Phycisphaerae; Phycisphaerales; SM1A02; JAEUIT01	4.28	62.58	288	96.21	0.57	32.7	3,735
						SAMN38186912	MP9P1.20	Bacteria; Verrucomicrobiota; Verrucomicrobiia	2.98	47.26	138	97.26	1.35	17.3	2,763
						SAMN38186913	MP9P1.49	Bacteria; Pseudomonadota; Alphaproteobacteria; Hyphomicrobiales; Rhodomicrobiaceae	3.11	57.39	84	98.59	0.4	35.7	3,115
						SAMN38186914	MP9P1.80	Bacteria; Bacteroidota; Bacteroidia; CAILMK01; CAILMK01	2.48	35.43	128	98.09	0	19.9	2,130
P2IB	SRX3539180	98	97	198,359	5291	SAMN38186915	P2IB.100	Bacteria; Pseudomonadota; Gammaproteobacteria; Lysobacterales; Ahniellaceae; 0-14-3-00-62-12	4.41	60.13	352	95.64	1.98	63.2	3,779
						SAMN38186916	P2IB.134	Bacteria; Planctomycetota; Planctomycetia; Pirellulales; PLA5	5.43	55.33	599	95.59	4.18	11.1	4,619
						SAMN38186917	P2IB.159	Bacteria; Bacteroidota; Bacteroidia; Flavobacteriales; UA16; UBA4660	3.63	45.16	89	99.73	0.29	62.8	3,161
						SAMN38186918	P2IB.169	Bacteria; Actinomycetota; Acidimicrobiia; IMCC26256; PALSA-610	3.6	68.4	395	96.41	3.02	16.1	3,701
						SAMN38186919	P2IB.181	Bacteria; Planctomycetota; Planctomycetia; Pirellulales; Pirellulaceae	7.3	51.16	310	95.22	1.18	31.4	5,757
						SAMN38186920	P2IB.185	Bacteria; Bacteroidota; Bacteroidia; Chitinophagales; Chitinophagaceae; JJ008	4.66	39.15	404	96.19	1.01	22.0	4,274
						SAMN38186921	P2IB.3	Bacteria; Acidobacteriota; Terriglobia; Bryobacterales; Bryobacteraceae; JACMLA01	7.5	61	456	98.48	0.87	17.3	6,778
						SAMN38186922	P2IB.85	Bacteria; Candidatus Hydrogenedentota; Hydrogenedentia; Hydrogenedentiales; SLHB01; JABWCD01	5.17	57	280	96.6	1.1	13.9	4,543
SP4G1	SRX3539186	97	96	182,794	4400	SAMN38186923	SP4G1.39	Bacteria; Bacteroidota; Bacteroidia; Chitinophagales; Chitinophagaceae; JJ008	4.42	41.53	224	99.51	0	17.7	3,896
						SAMN38186924	SP4G1.84	Bacteria; Pseudomonadota; Alphaproteobacteria; Hyphomicrobiales; Hyphomicrobiaceae; Hyphomicrobium aestuarii	3.69	61.99	258	95.35	2	17.9	3,472

^*a*^ Alternating grey and white bars indicate MAGs derived from the same metagenome.

These Antarctic MAGs help to remove barriers to researchers interested in the Antarctic by expanding the catalog of Antarctic bacterial genomes. They provide information that can be used to inform culturing efforts and be used in parallel with *in vitro* studies to understand how these organisms adapt to their extreme environment and may respond to ongoing climate change.

## Data Availability

Quality-controlled reads are available in the NCBI Sequence Read Archive under accession numbers SRR6448204–SRR6448219. Draft genomes are available under Genbank accession numbers JAWXAL01–JAWXCP01. Raw reads are available in IMG under project number Gs0127369.

## References

[1] Wade W. 2002. Unculturable bacteria—the uncharacterized organisms that cause oral infections. J R Soc Med 95:81–83. doi:10.1177/014107680209500207 11823550 PMC1279316

[2] Zhu D, Adebisi WA, Ahmad F, Sethupathy S, Danso B, Sun J. 2020. Recent development of extremophilic bacteria and their application in biorefinery. Front Bioeng Biotechnol 8:483. doi:10.3389/fbioe.2020.00483 32596215 PMC7303364

[3] Grettenberger CL, Sumner DY, Wall K, Brown CT, Eisen JA, Mackey TJ, Hawes I, Jospin G, Jungblut AD. 2020. A phylogenetically novel cyanobacterium most closely related to Gloeobacter. ISME J 14:2142–2152. doi:10.1038/s41396-020-0668-5 32424249 PMC7368068

[4] Sumner DY, Jungblut AD, Hawes I, Andersen DT, Mackey TJ, Wall K. 2016. Growth of elaborate microbial pinnacles in Lake Vanda, Antarctica. Geobiology 14:556–574. doi:10.1111/gbi.12188 27474373

[5] Bushnell B. 2018. BBtools. Sourceforge.Net/projects/Bbmap/

[6] Li D, Liu C-M, Luo R, Sadakane K, Lam T-W. 2015. MEGAHIT: an ultra-fast single-node solution for large and complex metagenomics assembly via succinct de Bruijn graph. Bioinformatics 31:1674–1676. doi:10.1093/bioinformatics/btv033 25609793

[7] Langmead B, Salzberg SL. 2012. Fast gapped-read alignment with Bowtie 2. Nat Methods 9:357–359. doi:10.1038/nmeth.1923 22388286 PMC3322381

[8] Li H, Handsaker B, Wysoker A, Fennell T, Ruan J, Homer N, Marth G, Abecasis G, Durbin R, 1000 Genome Project Data Processing Subgroup. 2009. The sequence alignment/map format and SAMtools. Bioinform Oxf Engl 25:2078–2079. doi:10.1093/bioinformatics/btp352 PMC2723002 19505943

[9] Kang DD, Froula J, Egan R, Wang Z. 2015. MetaBAT, an efficient tool for accurately reconstructing single genomes from complex microbial communities. PeerJ 3:e1165. doi:10.7717/peerj.1165 26336640 PMC4556158

[10] Parks DH, Imelfort M, Skennerton CT, Hugenholtz P, Tyson GW. 2015. CheckM: assessing the quality of microbial genomes recovered from isolates, single cells, and metagenomes. Genome Res 25:1043–1055. doi:10.1101/gr.186072.114 25977477 PMC4484387

[11] Jain C, Rodriguez-R LM, Phillippy AM, Konstantinidis KT, Aluru S. 2018. High throughput ANI analysis of 90K prokaryotic genomes reveals clear species boundaries. Nat Commun 9:5114. doi:10.1038/s41467-018-07641-9 30504855 PMC6269478

[12] Eren AM, Esen ÖC, Quince C, Vineis JH, Morrison HG, Sogin ML, Delmont TO. 2015. Anvi’o: an advanced analysis and visualization platform for ‘omics data. PeerJ 3:e1319. doi:10.7717/peerj.1319 26500826 PMC4614810

[13] Chaumeil P-A, Mussig AJ, Hugenholtz P, Parks DH. 2019. GTDB-TK: a toolkit to classify genomes with the genome taxonomy database. Bioinform Oxf Engl 36:1925–1927. doi:10.1093/bioinformatics/btz848 PMC7703759 31730192

[14] Lumian J, Sumner D, Grettenberger C, Jungblut AD, Irber L, Pierce-Ward NT, Brown CT. 2022. Biogeographic distribution of five Antarctic cyanobacteria using large-scale k-mer searching with sourmash branchwater. Biorxiv. doi:10.1101/2022.10.27.514113 PMC10909832 38440141

[15] Grettenberger C, Sumner DY, Eisen JA, Jungblut AD, Mackey TJ. 2021. Phylogeny and evolutionary history of respiratory complex I proteins in melainabacteria. Genes 12:929. doi:10.3390/genes12060929 34207155 PMC8235220

[16] Li W, O’Neill KR, Haft DH, DiCuccio M, Chetvernin V, Badretdin A, Coulouris G, Chitsaz F, Derbyshire MK, Durkin AS, Gonzales NR, Gwadz M, Lanczycki CJ, Song JS, Thanki N, Wang J, Yamashita RA, Yang M, Zheng C, Marchler-Bauer A, Thibaud-Nissen F. 2021. RefSeq: expanding the prokaryotic genome annotation pipeline reach with protein family model curation. Nucleic Acids Res 49:D1020–D1028. doi:10.1093/nar/gkaa1105 33270901 PMC7779008

